# PROF. PHD. ALCINO LÁZARO DA SILVA – FORMER PRESIDENT OF THE BRAZILIAN COLLEGE OF DIGESTIVE SURGERY

**DOI:** 10.1590/0102-672020220002e1691

**Published:** 2022-10-21

**Authors:** Paulo Roberto SAVASSI-ROCHA

**Affiliations:** 1 Universidade Federal de Minas Gerais – Belo Horizonte (MG), Brazil;; 2 Member and Former President of the Brazilian College of Digestive Surgery – São Paulo (SP), Brazil.

Santa Cruz da Prata was a tiny land between the mountains, close to the sky, in the south of the state of Minas Gerais (MG), Brazil. Also known as “Pratinha,” this region was once very beautiful, with sierras after sierras and woodlands with the Atlantic trees of Peroba, Jacarandá, Angelim, Angico, Ipês, and Sapucaias and all thousands of birds.

But there was a time when the coffee planters came; then, one by one, almost all trees were felled and, nowadays, very little, hardly anything, remains. What remained were those empty fields, the coffee plantations dominating a depleted landscape, outlining their dull uniformity. But, even today, when Spring arrives and the rains of September make the first buds bloom from the earth, the fields turn green and those magical mountains take over the silences and mysteries of MG.

It was there that, around 1930, João Biela da Silva, a respectable citizen and pharmacist, married Gentil Gianerini Silva, and they had five children: Antônio, Aldérico, Augusta, Alcino, and Ana Maria. To be more exact, our story begins on February 11, 1936, the date on which he, Alcino, was born.

It is difficult to distinguish history from story. Everything gets mixed up and combined after so many years that have gone. That is why I would like you to exempt me from eventual failures. As well stated by the Brazilian writer João Guimarães Rosa (Guimarães Rosa, *Grande Sertão: Veredas*, 1956, free translation): “You will please excuse this bad habit of mine. [...] I don’t know how to tell things straight. [...] Telling something is a very, very difficult business. Not because of the years that have gone by, but because certain things of the past have a way of changing about, switching places.”

All I know is that love and respect, brotherhood and discipline, work and faith reigned in that house of his. So many predicates created a fertile soil for a man with so many virtues to grow there. Furthermore, I know that, as he grew up, the family moved to Guaranésia, his birthplace, in the microregion of São Sebastião do Paraíso (MG), where Alcino completed his elementary education; then, the family moved to Mococa, in the northeast of the state of São Paulo, where he studied the first 2 years of high school education. As if the starts have aligned, once his vocation for Medicine was defined, he moved to Belo Horizonte (MG), where he completed high school at Colégio Marconi. Henceforth, he took the admission exam at the two Schools of Medicine then existing and was approved in both. He chose the Federal University (at the time called Universidade Minas Gerais — UMG), indisputably of better quality at the time, and began his brilliant career in Medicine in 1954. Ever since, and especially since his graduation in 1959, his life as a doctor, surgeon, and professor was an example to be followed. It translates into the nonconformity of a man who did everything to honor his profession and dignify it to the fullest extent. His connection with Prof. João Baptista de Resende Alves, then Full Professor of Surgical Technique and Experimental Surgery, from the fourth year of medicine onward, marked his academic and professional training, in such a way that, very early, he was endowed with the characteristics to become the natural heir of the direction of this exemplary surgical school.


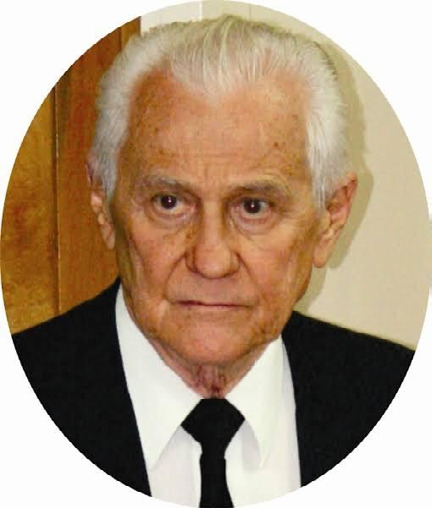


His stay in the municipality of Entre Rios de Minas lasted 7 years. There, together with his classmate Paulo Silva, he carried out one of the most beautiful works of all known to date. I made a point of personally visiting the city and the Cassiano Campolina Hospital, where he worked. I noticed, by listening to the testimonies of several people, that he did everything there: he organized the maternity ward, guided pregnant women, trained paramedics, provided free health care to the humble, plastered legs and arms, performed operations, and worked. The people there were grateful and granted him the honorable title of honorary citizen.

But his horizons were broader. Since then, even though he was settled in a small city, Professor Alcino would come to Belo Horizonte on weekends and spend hours on end at the Surgical Technique Laboratory, working on his doctoral dissertation, brilliantly defended in 1965.

At that time, he had already married Ana Maria, his wife, partner, friend, and his backbone for such an ambitious life project. Moreover, Ana gave him his greatest gifts: her love and their seven children.

I do not intend here to enumerate the Professor’s achievements and innumerable titles. The list is endless and the mission would be impossible in such a small space. So, as summarized as it could be, it would not do justice. However, as a matter of conscience, I feel obliged to report, through and through, some of his achievements since his definitive transfer to the University where he achieved the highest of all titles, that of Professor Emeritus. We should mention his hundreds of published works, his countless books, his brilliant civil service competitive examination for full professor in 1975, his extraordinary teaching participation, the creation of the Graduate Program in Surgery at the School of Medicine of Universidade Federal de Minas Gerais (UFMG), the coordination of numerous disciplines of undergraduate and graduate programs, and the countless honors received either as a sponsor lecturer or as a sponsoring professor of the classes^2^. It is also worth mentioning his efficient tenure as the president of the Brazilian College of Digestive Surgery (1997/1998)^1^, as the president of the Foundation for Research and Teaching in Surgery (FUPEC), at the Board of Directors of Hospital Borges da Costa, and the Brazilian Society for Surgery Research Development (SOBRADPEC), where he was the director. He was also, with all and indisputable merits, a member of the Minas Gerais Academy of Medicine and the National Academy of Medicine. He also received numerous honors such as the *Grande Medalha da Inconfidência* (Great Medal of Inconfidência), *Grande Medalha do Mérito da Saúde* (Great Medal of Merit in Health), and *Medalha da Ordem do Mérito Legislativo* (Medal of Legislative Merit), in addition to the honorable title of *Honoris Causa* Professor of Escola Superior da Santa Casa de Misericórdia de Vitória (state of Espírito Santo, Brazil). It is impossible for me not to say anything before his innovative and creative spirit, when devising surgical techniques that are now recognized worldwide, such as the proposal for the surgical correction of incisional hernias (Alcino Lazaro da Silva’s Technique)^3,4^. Finally, I cannot help but list the dozens of theses that the Professor has so wisely advised, the Digestive Surgery Service of Hospital das Clínicas, UFMG, which he created, among so many other activities^5^.

When he was the president of the Brazilian College of Digestive Surgery (1997/1998), he was responsible for acquiring its own headquarters. In addition, he was the one who included the Latin sentence “UT OMNES UNUM SINT,” which means “all for one,” in the College’s symbol, which has been maintained to this day^1^.

Words of aggrandizement and praise are usually a habit of those who greet the honoree. In my case, the praise is not just protocol-like: it is much rather a superior commandment of affection and recognition. Our points of view, at times divergent, have only increased my admiration, stimulated by my growing conviction of his greatness of character. Therefore, I allowed myself to trace his paths to show the saga of a man a little, as a humble and unnecessary attempt to frame him in the gallery of the great names of Brazilian surgery. Hence, I entered his backyards, wandered through his father’s pharmacy, tasted the flour cookies and guava sweets made by Jovita, a black mother, who today has a seat in heaven. I participated in his experiments in surgical technique, performed surgeries in Entre Rios, and scoured his enviable and immense curriculum, without any purpose.

At the end of my text, I sought to understand a fact that, at first, no one could answer me. I redoubled my efforts in tracing his steps, seeking to unravel his option to transfer to Belo Horizonte. I wondered why someone, living in the south of MG, near the border of São Paulo, and who, already studying in that state, decides to go to the capital of MG to study Medicine when, in those times, the most reasonable thing would be to choose the capital of São Paulo. I applied myself, with no support, to the search for this answer, so as to investigate and speculate on his life in a more accurate and competent way. Soon, that is what I found. I found the answer myself by knowing him, by tracing the enchanted universe of Guimarães Rosa (Guimarães Rosa, *Grande Sertão: Veredas*, 1956, free translation):

Those who are born in Minas Gerais are shy, benevolent, contained, suspicious, disciplined, discreet, scrupulous, thrifty, balanced, faithful, grateful, honorable, intelligent, honest, loyal, meditative, modest, obstinate, prudent, patient, mundane, sensible, without any haste, sagacious, sober, hardworking, timid, virtuous. People born in Minas Gerais bring more individuality than personality, they think that what matters is to be and not to appear, they know that to be agitated is not to act. Those born in Minas Gerais do not dispute. They have no visible audacity. They transcend it. They do not believe that anything can be solved by a gesture or an act, but they have learned that things come back, that life changes, that everything can come back.

When it dawned on me, I realized that Prof. Alcino had the face of MG and that there, only there, could he belong. In 2022, now enchanted, he left us a legacy that is difficult to fulfill.

Minas, our dear Minas, the “little homeland” of general stars.

Minas that comprises many: Jequitinhonha, Mata, Triângulo, Metalúrgica, Minas do Sul, and Minas of the western hills. Minas from all these cities, from Diamantina, Ouro Preto, Sabará, Cordisburgo, Lassance, Montes Claros, Entre Rios, Guaranésia, and Santa Cruz do Prata. Minas from Belo Horizonte.

Minas crossed by these rivers of deep waters, namely, the São Francisco, Grande, Doce, Paracatu, Carinhanha, Pandeiros, and the green-bluish Urucuia River, love of mine.

Minas that God carved and decorated with all the stones — the sapphires, tourmalines, amethysts, topaz — these golds of emerald hopes.

Minas, mountains, and sky and those mountain paths where Buriti trees touch the clouds.

Our Minas, ours alone. Minas of all Brazilians.

Minas that gave Brazil most of its greatest geniuses: Drumond, poet; Guimarães Rosa, the greatest of all writers; Santos Dumont who, one day, dreamed of flying like birds and did so; Carlos Chagas, the greatest scientist, who single-handedly discovered and fully described a disease; Pelé, who enchanted the world with his art; Tiradentes who, one day, died to free Brazil. Minas of Juscelino, Milton Campos, Mário Palmério, Hilton Rocha, Alfredo Balena, Baeta Vianna, and João Baptista de Resende Alves.

Minas of Alcino Lázaro da Silva.
